# A defined synthetic algal medium enables lettuce-free culturing of unfed *Paramecium bursaria* while preserving host-associated microbiome composition

**DOI:** 10.3389/fmicb.2026.1821058

**Published:** 2026-05-01

**Authors:** Yuri Matsushima, Eiko Himi, Masaharu Kitashima, Kohei Ogura, Susumu Kotani, Akiya Hino, Kazuhito Inoue, Hiroshi Hosoya

**Affiliations:** 1 Graduate School of Science, Kanagawa University, Yokohama, Kanagawa, Japan; 2 Faculty of Agriculture, Kibi International University, Minamiawaji, Hyogo, Japan; 3 Faculty of Chemistry and Biochemistry, Kanagawa University, Yokohama, Kanagawa, Japan; 4 Graduate School of Agriculture, Kyoto University, Uji, Kyoto, Japan; 5 Research Institute for Integrated Science, Kanagawa University, Yokohama, Kanagawa, Japan; 6 Tokyo Bunkyo Study Center, The Open University of Japan, Bunkyo, Tokyo, Japan

**Keywords:** *Chlorella variabilis*, ciliate, defined culture system, experimental reproducibility, microbiome, *Paramecium bursaria*, symbiosis, synthetic algal medium

## Abstract

*Paramecium bursaria* is widely cultured using undefined plant-based infusions such as lettuce extract, yet the variable composition of these media remains a major obstacle to experimental reproducibility and microbiome research. Here, we tested whether a chemically defined synthetic algal medium (AF-6) can replace conventional lettuce infusion while maintaining host physiology and associated microbial communities. An unfed clonal strain of *P. bursaria*, established in 2023 and capable of growth without external nutrient supplementation, proliferated comparably in AF-6 and lettuce media. To confirm that these results were not specific to unfed conditions, we additionally examined a publicly maintained algae-fed strain (NIES-2891), which exhibited similar growth patterns across both media. Cell size, compression-induced extension, and symbiotic algal abundance showed no significant differences between culture conditions. *rbc*L metataxonomic analysis revealed that *Chlorella variabilis* was the sole algal endosymbiont detected in all samples. Furthermore, 16S rRNA gene sequencing demonstrated that host-associated bacterial community composition remained largely conserved after replacement of lettuce infusion with AF-6 within each strain, although clear differences were observed between strains. Together, these findings establish an “unfed strain + defined algal medium” framework as a reproducible experimental platform for investigating tripartite interactions among ciliate hosts, symbiotic algae, and associated bacteria.

## 1 Introduction

The green ciliate *Paramecium bursaria* (*P. bursaria*) is a unicellular eukaryotic protist that harbors several hundred photosynthetic green algal symbionts within its cytoplasm. *P. bursaria* can proliferate not only by feeding on surrounding microorganisms such as bacteria but also by utilizing photosynthetic products provided by its intracellular symbiotic algae (Siegel, 1960; Karakashian, 1963; Reisser, 1988). Because both the host and the symbiotic partners are eukaryotic unicellular organisms, *P. bursaria* has long been regarded as a valuable experimental system for studying eukaryote–eukaryote symbiosis (Margulis and Bermudes, 1985; Fujishima and Kodama, 2012), and numerous studies have been conducted using this organism.

Notably, recent reassessments of experimental reproducibility have suggested that some earlier conclusions regarding *P. bursaria* may have been influenced by variability in culture conditions. Classical study has reported that symbiotic algae are not necessary for the maintenance of *P. bursaria* (Siegel, 1960). On the other hand, there are also reports that symbiotic algae are necessary for the maintenance of *P. bursaria* (Karakashian, 1963; Görtz, 1982; Fujishima and Kodama, 2012). Karakashian, in the paper in 1963, also argues that *P. bursaria* can be maintained without symbiotic algae in some cases. However, in each report, the composition of the culture medium is unclear and the bacteria used as nutrients are not consistent, making it difficult to compare and examine the reported findings.

Substantial variability has also been reported in growth rates depending on the culture medium used (Loefer, 1936; Wichterman, 1949). In many of these studies, culture media were prepared from natural sources such as lettuce extracts or pond water, resulting in considerable batch-to-batch variation in nutrient composition and associated microbial communities. Consequently, quantitative comparisons of growth and physiological properties across laboratories have been difficult to reproduce.

Taken together, these discrepancies are likely attributable to a lack of standardization in *P. bursaria* culture conditions among researchers. To improve experimental reproducibility and facilitate meaningful comparisons across studies, three major issues need to be addressed: (1) standardization or elimination of microorganisms added as food during cultivation, (2) use of cloned cells with defined and consistent genetic backgrounds, and (3) establishment of culture media with a chemically defined composition that can be shared among laboratories. To address the first two issues, we previously established multiple *P. bursaria* clones and selected a strain capable of continuous growth in sterilized lettuce medium without the addition of live bacteria or other microorganisms, thereby establishing an unfed strain (Himi et al., 2023).

Regarding the third issue, lettuce medium remains the most widely used culture medium for *P. bursaria* worldwide. However, because its composition depends on the physiological state and origin of lettuce leaves, researchers culturing *P. bursaria* in lettuce medium inevitably use media with different and undefined chemical compositions. This variability represents a major obstacle to standardization and reproducibility.

Therefore, the purpose of this study was to examine whether *P. bursaria* can be cultured using a synthetic medium with a defined chemical composition rather than a conventional medium of unknown composition. It has been established that symbiotic algae isolated from *P. bursaria* can be cultured independently and maintained using synthetic algal media commonly employed in algal research (Nishihara et al., 1998). Based on this knowledge, we hypothesized that *P. bursaria* itself might also be capable of sustained growth in synthetic algal media.

If *P. bursaria* can be reliably cultured using a combination of a defined synthetic algal medium and the unfed strain established in this study, this system would provide a standardized and reproducible experimental platform for investigating eukaryotic cell–cell symbiosis. Such standardization would greatly enhance the comparability of experimental results across laboratories and accelerate progress in symbiosis research using *P. bursaria*.

## 2 Materials and methods

### 2.1 Strains of *P. bursaria* and their culture conditions

We have reported the first unfed strains of *P. bursaria* (KUHH-3 and -4) (Himi et al., 2023). These strains had undergone three rounds of cloning by 2017 and have since been maintained under unfed conditions. In a previous study by Himi et al. (2023), fourth-round cloning was additionally conducted using the KUHH-3 and KUHH-4. For the present study, fourth-round cloning was again performed using the KUHH-4 in 2021 and a well growing clone was selected. This strain was designated as KUYO and used in this study. We further used the uncloned NIES-2891 strain, which was provided by the National Institute for Environmental Studies (NIES, Japan) as a representative of conventional strains that require feeding in culture. This NIES-2891 strain has been continuously maintained at NIES under algal feeding conditions.

These strains (300–500 cells/mL) were maintained in lettuce medium prior to the experiment. For culturing *P. bursaria*, 3 mL/well of medium was placed in a 6-well plate (TPP, 92006), and a small volume of the culture was transferred into fresh lettuce or AF-6 medium to achieve an initial density of approximately 10 cells/mL. Cells were cultured at 23 °C in an incubator (Biotron, LH-411S) under light conditions (12 h light/12 h dark, 50–60 μmol photons m^–2^ s^–1^) without administering exogenous microorganisms for the unfed strain or in the presence of exogenous symbiotic algae for NIES strain. The number of *P. bursaria* cells was counted every few days. For culturing, lettuce medium and algal medium (AF-6) were used.

### 2.2 *P. bursaria* counting method

For total count, all *P. bursaria* in the well (3 mL) were counted. When *P. bursaria* multiplied and it became difficult to count the total population, grid count was performed. For grid count, the 6-well plate was placed on transparent graph paper with fine grids of 5 or 2.5 mm squares, and *P. bursaria* in 10 grids were counted under a microscope. The total number of *P. bursaria* in the well was calculated by multiplying this value by the bottom area of one well divided by the area of 10 grids.

### 2.3 Medium preparation

Lettuce medium was prepared according to Matsushima et al. (2022) using lettuce leaves, autoclaved, and stored in a refrigerator. Synthetic algal medium AF-6 was obtained from the NIES. AF-6 medium was prepared following the formulation of the NIES-Collection, which is based on the original AF-6 medium described by Kato (1982), with modifications including the use of PIV metal solution and buffering with MES (Watanabe et al., 2000).

### 2.4 Measurement of body size and number of symbiotic algae in *P. bursaria* cells

Culture solution (16.2 μL) was placed on a glass slide and covered with a cover glass (Matsunami, 18 mm × 18 mm). The swimming cells were photographed and their body size was measured from the images. We also measured the body size of cells that were on the verge of bursting under pressure from the cover glass, and calculated the cell elongation rate from “body size on the verge of bursting ÷ body size during swimming.” The autofluorescence of symbiotic algae leaked from the ruptured cells was observed, and symbiotic algae were counted. All of these experiments were independently performed in triplicate for each strain. Statistical comparisons of body size or number of symbiotic algae between media conditions were conducted using Student’s *t*-test.

### 2.5 Species identification of symbiotic algae

The *P. bursaria* cells were collected by centrifugation at 4,500 × *g* for 5 min and disrupted using Lysis Solution F (Nippon Gene, Tokyo, Japan) and Shake Maker Neo (Bio Medical Science, Tokyo, Japan). After 10 min incubation at room temperature, supernatant was collected by centrifugation 12,000 × *g* for 2 min, followed by DNA extraction using Lab-Aid824s DNA Extraction kit (ZEESAN, Fujian, China). For library preparation of *rbc*L sequences, PCR was conducted with the pair of primers: Fw_*rbc*L_192, 5′- ACACTCTTTCCCTACACGACGCTCTTCCGATCT-GGTACTTGGACAACWGTWTGGAC-3′ and Rv_*rbc*L_657, 5′-GTGACTGGAGTTCAGACGTGTGCTCTTCCGATCT-GAAACGGTCTCKCCARCGCAT-3′with adapter sequences. Barcode sequences were added in the second PCR. Two rounds of PCR and amplicon sequencing were performed on the MiSeq system (Illumina, CA, United States). The 300 bp pair-end reads were processed by FASTX-Toolkit (ver. 0.0.14)^1^, followed by removal of reads with QC below 20 by sickle (ver. 1.33)^2^. The pair-ends were merged by FLASH (ver. 1.2.11) with a minimum overlap of 10 bases (Magoč and Salzberg, 2011). After removal of noise and chimera sequences by DADA2 plug-in (Callahan et al., 2016), nucleotide BLAST (ver. 2.13.0) was conducted using the representative sequences obtained in Qiime2 programs (ver. 2023.7) as queries against NCBI nucleotide database (Altschul et al., 1990). The sequence analysis was outsourced to the Bioengineering Lab. Co. Ltd. (Sagamihara, Kanagawa, Japan).

### 2.6 Bacterial 16S rRNA gene analysis

The microbiome was analyzed according to a previous report (Himi et al., 2023) with slight modifications. For library preparation of 16S rRNA V3-V4 regions, PCR was conducted with the pair of primers 341f (5′-ACACTCTTTCCCTACACGACGCTCTTCCGATCT-NNNNN-CCTACGGGNGGCWGCAG-3′) and 805r (5′-GTGACTGGAGTTCAGACGTGTGCTCTTCCGATCT-NNNNN-GACTACHVGGGTATCTAATCC-3′). After addition of the adaptor and barcode sequences, the library was prepared using MiSeq Reagent Kit v3 (Illumina) and sequenced by MiSeq system. The 300 bp pair-end reads were applied to FASTX-Toolkit (ver. 0.0.14) to remove non-specific amplicons and the primer sequences, followed by merge of the pair-end reads by FLASH (ver. 1.2.11). The merged reads were analyzed using Qiime2 programs (Bolyen et al., 2019). Noise and Chimera sequences were removed by the DADA2 plug-in (Callahan et al., 2016). The representative sequences of amplicon sequence variants (ASVs) were classified using machine-learned classifier created using Greengenes (ver. 13_8) database (DeSantis et al., 2006). Taxonomic levels shown in Figure 3 include genus, family, and order ranks, reflecting differences in classification resolution among ASVs assigned by the Greengenes-based classifier. Correlation analysis was performed using LEfSe package in R (ver. 4.1.2), and sequence analysis was outsourced to the Bioengineering Lab. Co. Ltd. For analysis of alpha and betta diversity, diversity metrics were calculated at a rarefaction depth of 20,000 reads per sample.

## 3 Results

### 3.1 Growth of *P. bursaria* unfed strain

**FIGURE 1:**
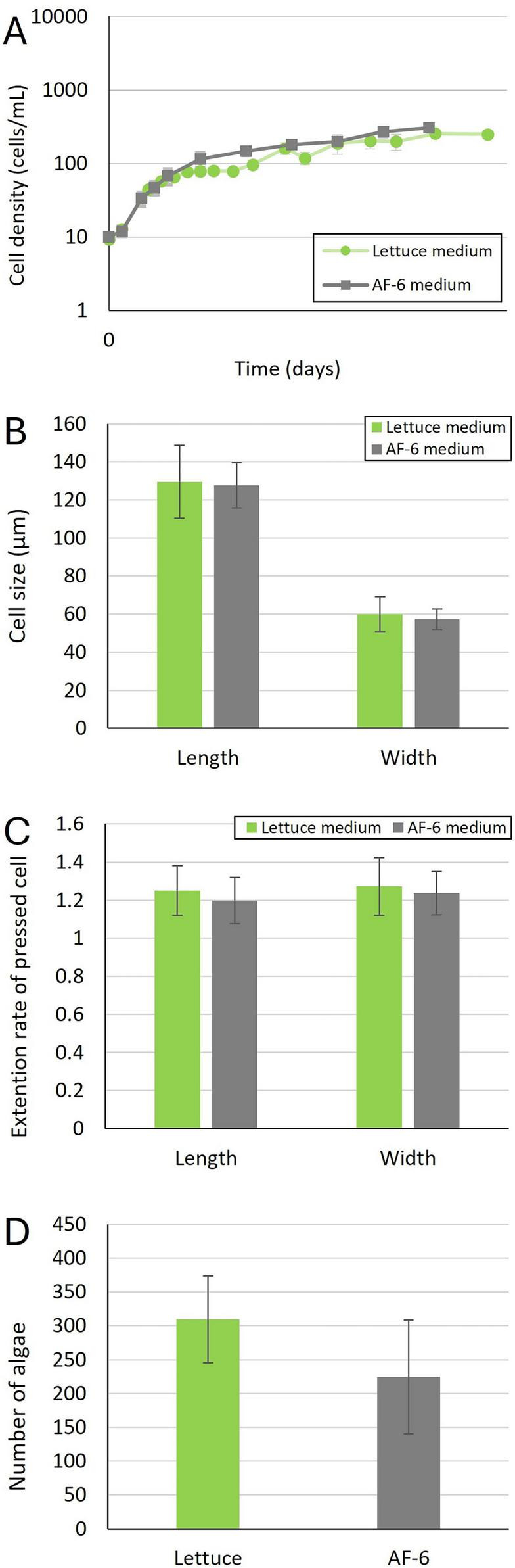
Physiological characteristics of the unfed *P. bursaria* strain. **(A)** Cell proliferation of the unfed *P. bursaria* strain in different culture media. Data represent the mean values from three independent experiments, with error bars indicating standard deviations. **(B)** Cell size of the unfed *P. bursaria* strain during swimming. Mean values were calculated from measurements of 12 cells (four cells from each of three independent experiments cultured under identical conditions). Error bars indicate standard deviations. **(C)** Cell extension rates of the unfed *P. bursaria* strain. Mean values were obtained from measurements of 12 cells, consistent with the conditions described in panel **(B)**. Error bars represent standard deviations. **(D)** Number of symbiotic algae per unfed *P. bursaria* strain cell. Mean values were calculated from six cells (two cells from each of three independent experiments cultured under the same conditions). Error bars indicate standard deviations.

To investigate whether unfed *P. bursaria* strain can grow in synthetic algal medium, we compared the growth in lettuce medium and synthetic algal medium (AF-6). The strain displayed similar growth curves between the two media in three times independent experiments (Figure 1A).

### 3.2 Properties of unfed *P. bursaria* strain

We investigated changes in cell morphology (cell length and width sizes) and the numbers of symbiotic algae in the two media. Statistical analysis using Student’s *t*-test detected no significant differences in the size of the long and short axes (Figure 1B), the degree of cell extension after compression (Figure 1C), or the number of symbiotic algae (Figure 1D) between the two types of media. These data confirmed that synthetic algal medium is useful for culturing unfed *P. bursaria* strains.

### 3.3 Growth of NIES strain, an uncloned and publicly maintained *P. bursaria* strain

**FIGURE 2:**
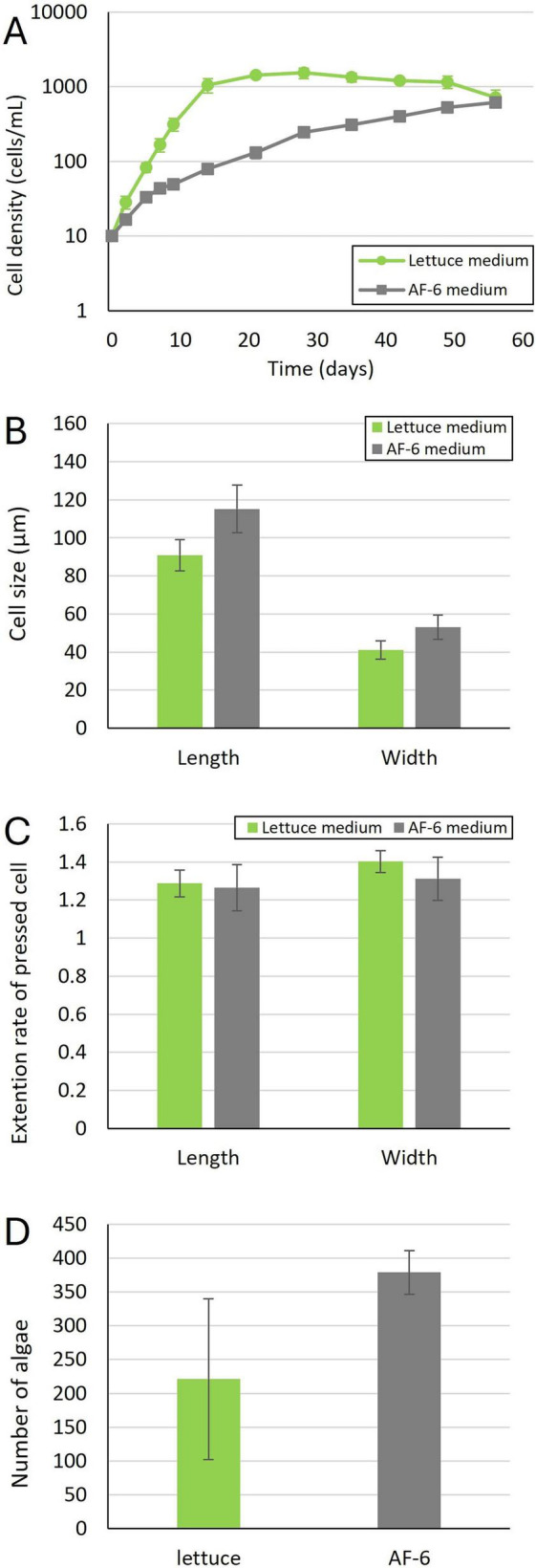
Physiological characteristics of the *P. bursaria* National Institute for Environmental Studies (NIES). **(A)** Cell proliferation of the *P. bursaria* NIES in different culture media. Data represent the mean values from three independent experiments, with error bars indicating standard deviations. **(B)** Cell size of the *P. bursaria* NIES during swimming. Mean values were calculated from measurements of 12 cells (four cells from each of three independent experiments cultured under identical conditions). Error bars indicate standard deviations. **(C)** Cell extension rates of the *P. bursaria* NIES. Mean values were obtained from measurements of 12 cells, consistent with the conditions described in panel **(B)**. Error bars represent standard deviations. **(D)** Number of symbiotic algae per *P. bursaria* NIES cell. Mean values were calculated from six cells (two cells from each of three independent experiments cultured under the same conditions). Error bars indicate standard deviations.

Next, we analyzed growth of an uncloned strain that requires the feeding of algae as a nutrient source during cultivation (NIES strain in this study), which has been widely available for many researchers. We cultured this NIES strain in the two media, lettuce medium and AF-6. As the results, NIES strain also grew well not only in a lettuce medium but also in an AF-6 medium (Figure 2A).

**FIGURE 3:**
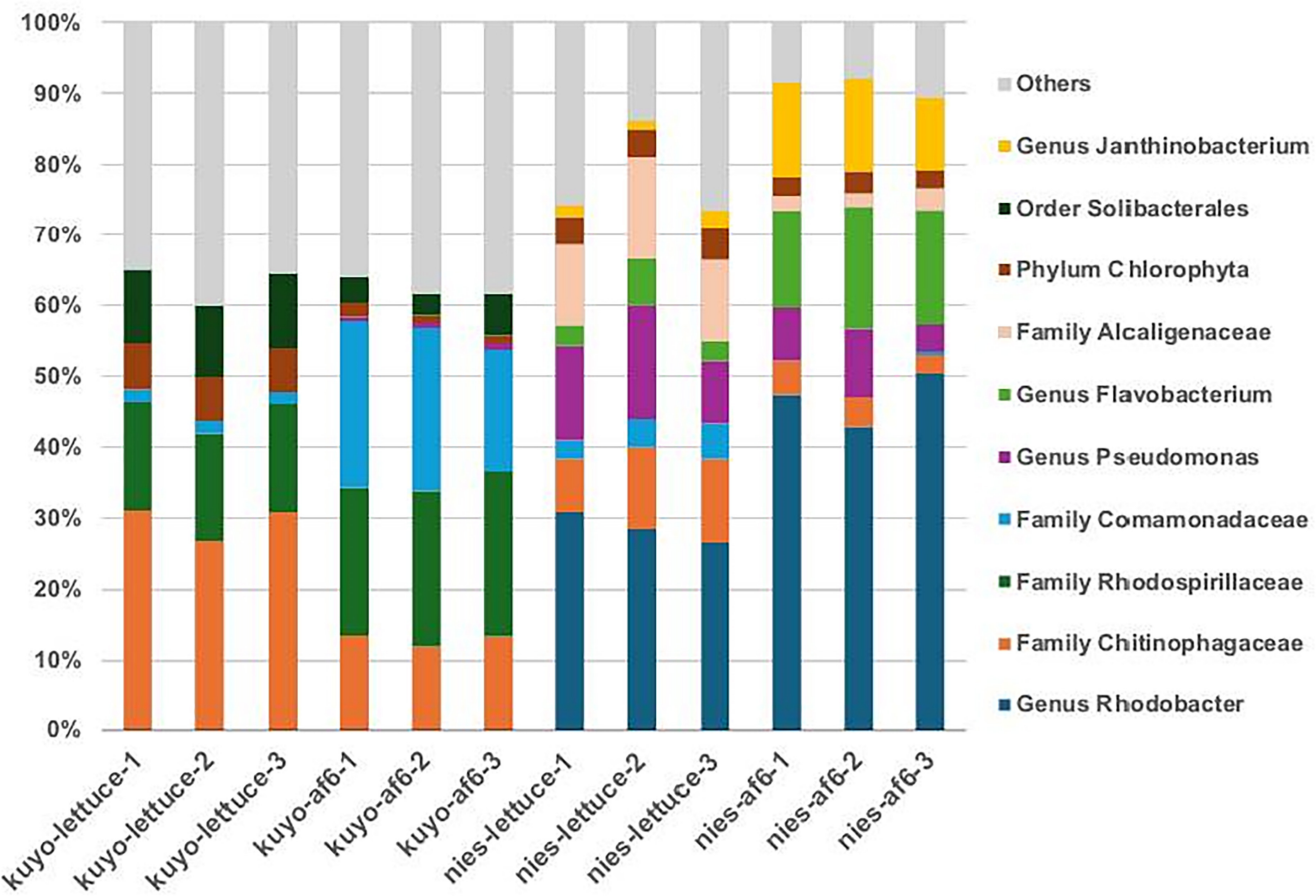
Relative abundance of the top 10 taxonomic groups (genus, family, or order level depending on classification resolution) detected across all 12 samples in *P. bursaria* unfed strain (KUYO) and National Institute for Environmental Studies (NIES). Stacked bar plots showing the composition of the most dominant taxonomies including genera, family, phylum and order based on the assigned amplicon sequence variants (ASVs). The metataxonomic data were shown in Supplementary Table S2.

### 3.4 Properties of *P. bursaria* NIES strain

We also examined whether changes in morphology (cell length and width) and the number of symbiotic algae occurred in NIES cells cultured in each medium. The length and width of cells cultured in AF-6 medium tended to be slightly larger than those cultured in lettuce medium (Figure 2B). No significant differences were observed in the degree of cell extension after compression between the two media (Figure 2C). The number of symbiotic algae within the cells varied among NIES cells in lettuce medium, but remained stable in AF-6 medium (Figure 2D). These results demonstrate that the NIES strain can be also cultured in both lettuce and algal medium.

### 3.5 Identification of symbiotic algae species

We compared the differences of the intracellular symbiotic algae between the unfed strain and the NIES strain. The symbiotic algae were typed based on amplicon sequences of the *rbc*L genes. For the unfed strains, three samples were prepared for cultivation in lettuce medium and another three for cultivation in AF-6 medium, and *P. bursaria* was grown in all six samples. The same cultivation procedure was applied to the NIES strains. Subsequently, *rbcL* gene analysis was performed on all 12 samples. As a result, *Chlorella variabilis*, a representative symbiotic algae strain of *P. bursaria*, dominated nearly 100% of all the 12 samples. This suggests that the symbiotic algae in *P. bursaria* were commonly dominated by *C. variabilis* regardless of culture conditions or nutritional requirements (Supplementary Table S1).

### 3.6 Identification of bacterial compositions

Amplicon sequencing of the V3–4 region of the 16S rRNA gene was used to analyze the bacterial composition of the *P. bursaria* unfed and NIES strains (Supplementary Table S2). The bacterial compositions were clearly different between the unfed and NIES strains (Figure 3). These markedly different community structures in their bacterial compositions were also confirmed by quantitative correlation analysis (Supplementary Table S3). Regarding the individual bacteria detected in each strain, the NIES strain contained bacteria of various cloors, while the unfed strain did not.

Next, we compared how the bacterial composition changed on different media, lettuce medium and AF-6 medium. As shown in Figure 3 and Supplementary Table S3, no significant changes in bacterial composition were observed between the two media. Quantitative correlation analysis also revealed high similarity in their bacterial compositions. For both strains of *P. bursaria*, the type of medium was shown to have only a slight effect on the bacterial composition (Supplementary Table S3).

To further evaluate bacterial community structure, we performed additional diversity analyses (Supplementary Figure S1). Alpha diversity analysis showed no significant differences between lettuce and AF-6 media in the KUYO strain for either the Shannon index or Faith’s phylogenetic diversity. A modest decrease in Shannon diversity was observed in the NIES strain cultured in AF-6, whereas Faith’s phylogenetic diversity remained unchanged.

Beta diversity analysis using PCoA based on Bray–Curtis distances revealed clear separation between KUYO and NIES samples (*P* = 0.049 by PERMANOVA), whereas differences between media were relatively small (*p* > 0.10).

## 4 Discussion

### 4.1 *P. bursaria* proliferates in the synthetic algal medium

As shown in Figures 1, 2, the unfed and NIES strains of *P. bursaria* used in this experiment grew well in AF-6 medium, a common synthetic culture medium used for algae cultivation. However, the growth rate of the NIES strain during the logarithmic growth phase tended to be slower in AF-6 medium compared to lettuce medium. Although a small amount of pre-culture lettuce medium may be introduced during inoculation in AF-6, the sustained growth observed in AF-6 suggests that the defined medium itself is sufficient to support *P. bursaria* proliferation.

In addition, it was found that there was no significant difference in the size of both *P. bursaria* grown in AF-6 medium and lettuce medium, and in the number of symbiotic algae in the paramecium cells.

Relative changes in cell size before and after pressure application provide an indirect indicator of the overall mechanical resistance of the cells (Fletcher and Mullins, 2010). As shown in Figures 1C, 2C, the rate of cell size change upon pressure application did not differ between the two media or between the two strains. This finding suggested that the apparent global mechanical resistance of *P. bursaria* cells is comparable under both culture conditions.

Although we cannot completely exclude the possibility that intracellularly stored nutrients contribute to initial growth after transfer, the sustained proliferation observed over time indicates that AF-6 medium supports continued growth beyond any transient effects of pre-culture conditions.

Taken together, these results strongly indicate that synthetic algal media such as AF-6 can serve as a reliable alternative to lettuce medium for culturing *P. bursaria*, enabling more standardized culture conditions in future research.

### 4.2 Symbiotic algal composition in *P. bursaria* is the same on lettuce and synthetic algal media

As mentioned above, the number of symbiotic algae in unfed *P. bursaria* strains and NIES grown on synthetic algal medium did not differ from that observed in lettuce medium (Figures 1D, 2D). To identify the symbiotic algal species, we performed amplicon sequencing using primers targeting a specific region of the *rbc*L gene. The algal endosymbionts of *P. bursaria* are known to encompass several species within Chlorellaceae, most commonly *C. variabilis*, although *Micractinium conductrix*, *C. vulgaris*, and *C. sorokiniana* were also reported depending on host syngen and geographic origin. This pattern is supported by recent phylogeographic surveys and diagnostic PCR studies (Spanner et al., 2020, 2022; Greczek-Stachura et al., 2021).

The use of *rbc*L amplicon sequencing is well-suited for distinguishing among closely related Chlorellaceae species, as the *rbc*L locus provides sufficient phylogenetic resolution to discriminate *C. variabilis*, *M. conductrix*, and other reported endosymbionts of *P. bursaria*. However, as shown in Supplementary Table S1, our *rbc*L-based analysis consistently detected only *C. variabilis* across all conditions tested. This uniformity suggested that the unfed and NIES strains examined here maintain a stable and specific symbiotic association that is not influenced by the type of culture medium. *Micractinium reiserri*, previously reported by Hoshina et al. (2010) and often referred to as *M. conductrix* in earlier literature, was also not detected. The absence of *M. reiserri* further indicates that neither AF-6 nor lettuce medium induces a shift in algal symbiont identity. Instead, the symbiotic partnership appears robust to environmental variation in nutrient composition, at least within the range of media tested.

### 4.3 Bacterial composition in *P. bursaria* is similar on lettuce and synthetic algal media

Interestingly, during the process of establishing a *P. bursaria* unfed strain, bacteria have been always detected in the medium of the unfed strain. The unfed strain did not grow in the presence of antibiotics, suggesting that the presence of bacteria is essential for the growth of *P. bursaria* (Himi et al., 2023). In fact, 16S rDNA sequence analysis further revealed that the unfed strain contained multiple species of bacteria with bioremediation and plant growth-promoting activity (Himi et al., 2023). The study has suggested that although the established strain is clonal, growth rates can still vary among individuals, and this diversity depends on the type of bacteria detected in each individual. For example, in fast-growing individuals, rhizobia of the family *Rhodospirillaceae* and *Xanthobacter* bacteria were dominant. *Rhizobia* are known to have the ability to fix atmospheric nitrogen and exhibit high plant growth-promoting activity, and are presumed to be the main bacteria promoting the growth of unfed *P. bursaria* strains (Himi et al., 2023).

Therefore, we also performed amplicon sequencing of bacteria present in various *P. bursaria* grown in synthetic algal media to examine whether differences in bacterial composition were observed between the media. The results in Figure 3 show that for the unfed strain, common bacteria were detected when it was cultured on both lettuce medium and synthetic algal medium. Similar results were obtained for the NIES-2891. These facts indicate that there is no significant change in the bacterial composition in the cells of *P. bursaria*, even when the media is different. A detailed analysis of the bacterial composition revealed that both of the unfed strain and NIES-2891 contained family *Chitinophagaceae*. Abundance ratios of the family were significantly lower in the AF-6 cultured groups than the lettuce-fed groups of both unfed strains (*P* = 0.007 by Welch’s *t*-test) and NIES-2891 (*P* = 0.024). Although the results suggested that some nutrition changes the abundance ratio of family *Chitinophagaceae*, it remains unknown which compound is involved or which genera or species in the family was affected. In the NIES-2891, abundance ratios of genera *Rhodobacter*, *Flavobacterium*, and *Janthinobacterium* were significantly higher in the AF-6 fed group with *P*-values of 0.005, 0.002, and 0.001, respectively. Nonetheless, the correlation between the different media was very high, suggesting that the effect of the media on bacterial flora composition was considered small. These results suggest that host-associated bacterial communities are primarily determined by host lineage rather than by basal culture medium composition.

These observations were further supported by community-level diversity analyses. While a modest decrease in Shannon diversity was observed in the NIES strain under AF-6 conditions (Supplementary Figure S1A), no corresponding change was detected in Faith’s phylogenetic diversity, suggesting that medium-dependent effects are limited to shifts in relative abundance rather than major changes in phylogenetic composition. Moreover, beta diversity analysis demonstrated that bacterial communities were clearly separated according to host strain (Supplementary Figure S1B), indicating that host lineage exerts a stronger influence on microbiome structure than the basal culture medium composition. Although the present study did not address temporal changes in bacterial population dynamics during host growth, such analyses will be essential to elucidate the functional roles of associated bacteria in *P. bursaria.* The defined culture system established here provides a suitable experimental framework for such future investigations.

### 4.4 The bacterial composition varies greatly among two kinds of *P. bursaria*

The bacterial composition differed significantly between the two types of *P. bursaria* (Supplementary Table S2). This suggests that each strain of *P. bursaria* has the unique bacteria moving in and out of *P. bursaria* (sometimes as symbiont). A detailed analysis revealed that the unfed strains primarily contained the families *Chitinophagaceae*, *Rhodospirillaceae*, and *Comamonadaceae*, although their subranks (genera) were not assigned. In contrast, the NIES were dominated by the genera *Rhodobacter*, *Pseudomonas*, *Flavobacterium*, and *Janthinobacterium*. *Rhodobacter phaeroides*, one of the major *Rhodobacter* species, is a purple photosynthetic bacterium (Kiley and Kaplan, 1988). *Janthinobacterium* species produce a purple pigment called violacein involved in oxidant tolerance (Park et al., 2021). *Flavobacterium* species exhibit various colors such pale, yellow, or their mixed colors due to production of two pigments carotenoid and flexirubin (Whitman et al., 2015). It remains unclear why NIES, but not unfed strains, contained these pigment -producing bacteria.

### 4.5 Implications for reproducibility and standardization of *P. bursaria* culture systems

Notably, culture practices for *P. bursaria* vary widely among laboratories, particularly with respect to feeding microorganisms and basal media. Different researchers provide diverse nutritional sources, including bacteria such as *Klebsiella pneumoniae* or phototrophic microorganisms such as *Chlorogonium elongatum* (Berk et al., 1991; Sonneborn, 1970; Steinbrück et al., 1981; Görtz, 1982; Omura et al., 2004; Greczek-Stachura et al., 2021). Lettuce medium remains widely used (Barna and Weis, 1973; Weis, 1975), whereas hay infusion or tryptone-based media are also employed in some laboratories (Wichterman, 1949; Weis, 1975; Görtz, 1982). Because these approaches differ markedly in chemical composition and microbial inputs, reproducible cross-laboratory comparisons of growth, symbiont dynamics, and host-associated bacteria remain difficult.

The present study provides a practical route to mitigate this long-standing problem. We show that a defined synthetic algal medium (AF-6) supports stable proliferation of *P. bursaria* while maintaining symbiotic algal identity and preserving gross cellular properties. Importantly, within each strain, bacterial community composition remained largely conserved after switching from lettuce to AF-6, suggesting that adoption of defined media does not necessarily disrupt host-associated bacteria. Together with our previously established unfed clonal strain (Himi et al., 2023), these results support the feasibility of reducing reliance on variable external feeding regimes while adopting a chemically defined medium as a shared baseline condition.

Rather than proposing a single universal protocol, we suggest that an “unfed strain + defined algal medium” framework can serve as a reference culture system to improve reproducibility and facilitate direct comparisons among laboratories. Such standardization may enable more rigorous microbiological investigations of eukaryote–eukaryote symbiosis alongside host-associated microbiomes in *P. bursaria*.

## 5 Conclusion

In conclusion, we demonstrate that the defined synthetic algal medium AF-6 can replace conventional lettuce infusion for culturing unfed *P. bursaria*. Both an unfed clonal strain (KUYO) and a publicly maintained, algae-fed strain (NIES-2891) proliferated comparably in AF-6 and lettuce media, with no major differences in cell size, compression-induced extension, or symbiont load. *rbc*L metataxonomic analysis identified *C. variabilis* as the sole algal endosymbiont across all conditions tested. Importantly, host-associated bacterial community composition was largely conserved after switching from lettuce infusion to AF-6 within each strain, whereas marked differences were observed between strains. Together, these findings establish an “unfed strain + defined algal medium” framework as a reproducible baseline culture system for investigating tripartite interactions among the ciliate host, algal symbionts, and associated bacteria.

## Data Availability

The datasets presented in this study can be found in online repositories. The names of the repository/repositories and accession number(s) can be found in the article/Supplementary material.

## Footnotes

1
http://hannonlab.cshl.edu/fastx_toolkit

2
https://github.com/najoshi/sickle

## References

[B1] Altschul S. F. Gish W. Miller W. Myers E. W. Lipman D. J. (1990). Basic local alignment search tool. *J. Mol. Biol.* 215 403–410. 10.1016/S0022-2836(05)80360-2 2231712

[B2] Barna I. Weis D. S. (1973). The utilization of bacteria as food for *Paramecium bursaria*. *Trans. Am. Microsc. Soc.* 92 434–440. 10.2307/3225247 4199960

[B3] Berk S. G. Parks L. H. Ting R. S. (1991). Photoadaptation alters the ingestion rate of *Paramecium bursaria*, a mixotrophic ciliate. *Appl. Environ. Microbiol.* 57 2312–2316. 10.1128/aem.57.8.2312-2316.1991 16348540 PMC183569

[B4] Bolyen E. Rideout J. R. Dillon M. R. Bokulich N. A. Abnet C. C. Al-Ghalith G. A. et al.. (2019). Reproducible, interactive, scalable and extensible microbiome data science using QIIME 2. *Nat. Biotechnol.* 37 852–857. 10.1038/s41587-019-0209-9 31341288 PMC7015180

[B5] Callahan B. J. McMurdie P. J. Rosen M. J. Han A. W. Johnson A. J. A. Holmes S. P. (2016). DADA2: High-resolution sample inference from Illumina amplicon data. *Nat. Methods* 13 581–583. 10.1038/nmeth.3869 27214047 PMC4927377

[B6] DeSantis T. Z. Hugenholtz P. Larsen N. Rojas M. Brodie E. L. Keller K. et al.. (2006). Greengenes, a chimera-checked 16S rRNA gene database and workbench compatible with ARB. *Appl. Environ. Microbiol.* 72 5069–5072. 10.1128/AEM.03006-05 16820507 PMC1489311

[B7] Fletcher D. A. Mullins R. D. (2010). Cell mechanics and the cytoskeleton. *Nature* 463 485–492. 10.1038/nature08908 20110992 PMC2851742

[B8] Fujishima M. Kodama Y. (2012). Endosymbionts in paramecium. *Eur. J. Protistol.* 48 124–137. 10.1016/j.ejop.2011.10.002 22153895

[B9] Görtz H. D. (1982). Infections of *Paramecium bursaria* with bacteria and yeasts. *J. Cell Sci.* 58 445–453. 10.1242/jcs.58.1.445 7183698

[B10] Greczek-Stachura M. Leśnicka P. Z. Tarcz S. Rautian M. Możdżeń K. (2021). Genetic diversity of symbiotic green algae of *Paramecium bursaria* syngens originating from distant geographical locations. *Plants* 10:609. 10.3390/plants10030609 33806926 PMC8005025

[B11] Himi E. Miyoshi-Akiyama T. Matsushima Y. Shiono I. Aragane S. Hirano Y. et al.. (2023). Establishment of an unfed strain of *Paramecium bursaria* and analysis of associated bacterial communities controlling its proliferation. *Front. Microbiol.* 14:1036372. 10.3389/fmicb.2023.1036372 36960277 PMC10029143

[B12] Hoshina R. Iwataki M. Imamura N. (2010). *Chlorella variabilis* and *Micractinium reisseri* sp. nov. (Chlorellaceae, Trebouxiophyceae): Redescription of the endosymbiotic green algae of *Paramecium bursaria* (Peniculia, Oligohymenophorea) in the 120th year. *Phycol. Res.* 58 188–201. 10.1111/j.1440-1835.2010.00579.x

[B13] Karakashian S. J. (1963). Growth of *Paramecium bursaria* as influenced by the presence of algal symbionts. *Physiol. Zool.* 36 52–68. 10.1086/physzool.36.1.30152738

[B14] Kato S. (1982). Laboratory culture and morphology of *Colacium vesiculosum* Ehrb. (Euglenophyceae). *Jpn. J. Phycol.* 30 63–67.

[B15] Kiley P. J. Kaplan S. (1988). Molecular genetics of photosynthetic membrane biosynthesis in *Rhodobacter sphaeroides*. *Microbiol. Rev.* 52 50–69. 10.1128/mr.52.1.50-69.1988 3280966 PMC372705

[B16] Loefer J. B. (1936). Bacteria-free culture of *Paramecium bursaria* and concentration of the medium as a factor in growth. *J. Exp. Zool*. 72 387–407. 10.1002/jez.1400720303

[B17] Magoč T. Salzberg S. L. (2011). FLASH: Fast length adjustment of short reads to improve genome assemblies. *Bioinformatics* 27 2957–2963. 10.1093/bioinformatics/btr507 21903629 PMC3198573

[B18] Margulis L. Bermudes D. (1985). Symbiosis as a mechanism of evolution: Status of cell symbiosis theory. *Symbiosis* 1 101–124. 11543608

[B19] Matsushima Y. Hirano Y. Iwanaga M. Komiya M. Kondo N. Ominato Y. et al.. (2022). Establishment of a method tao culture a washed and cloned green *Paramecium* (*Paramecium bursaria*). *Sci. J. Kanagawa Univ.* 33 1–4.

[B20] Nishihara N. Horiike S. Takahashi T. Kosaka T. Shigenaka Y. Hosoya H. (1998). Cloning and characterization of endosymbiotic algae isolated from *Paramecium bursaria*. *Protoplasma* 203 91–99. 10.1007/BF01280591

[B21] Omura G. Ishida M. Arikawa M. Mostafa Kamal K. S. M. Suetomo Y. Kakuta S. et al.. (2004). A bacteria-free monoxenic culture of *Paramecium bursaria*: Its growth characteristics and the re-establishment of symbiosis with Chlorella in bacteria-free conditions. *Jpn. J. Protozool.* 37 139–150. 10.18980/jjprotozool.37.2_139

[B22] Park H. Park S. Yang Y.-H. Choi K.-Y. (2021). Microbial synthesis of violacein pigment and its potential applications. *Crit. Rev. Biotechnol.* 41 879–901. 10.1080/07388551.2021.1892579 33730942

[B23] Reisser W. (1988). “Signals in the *Paramecium bursaria* — Chlorella Sp. — association,” in *Cell to Cell Signals in Plant, Animal and Microbial Symbiosis*, eds Scannerini S. Smith D. Bonfante-Fasolo P. Gianinazzi-Pearson V. (Berlin: Springer), 271–282.

[B24] Siegel R. W. (1960). Hereditary endosymbiosis in *Paramecium bursaria*. *Exp. Cell Res.* 19 239–252. 10.1016/0014-4827(60)90005-7 14446477

[B25] Sonneborn T. M. (1970). “Chapter 12 methods in *Paramecium* research,” in *Methods in Cell Biology*, ed. Prescott D. M. (Amsterdam: Elsevier), 241–339.

[B26] Spanner C. Darienko T. Biehler T. Sonntag B. Pröschold T. (2020). Endosymbiotic green algae in *Paramecium bursaria*: A new isolation method and a simple diagnostic PCR approach for the identification. *Diversity* 12:240. 10.3390/d12060240

[B27] Spanner C. Darienko T. Filker S. Sonntag B. Pröschold T. (2022). Morphological diversity and molecular phylogeny of five *Paramecium bursaria* (Alveolata, Ciliophora, Oligohymenophorea) syngens and the identification of their green algal endosymbionts. *Sci. Rep.* 12:18089. 10.1038/s41598-022-22284-z 36302793 PMC9613978

[B28] Steinbrück G. Haas I. Hellmer K. H. Ammermann D. (1981). Characterization of macronuclear DNA in five species of ciliates. *Chromosoma* 83 199–208. 10.1007/BF00286789 6791900

[B29] Watanabe M. M. Kawachi M. Hiroki M. Kasai F. (2000). *NIES-Collection. List of Strains: Microalgae and Protozoa*, 6 Edn. Tsukuba: National Institute for Environmental Studies.

[B30] Weis D. S. (1975). A medium for the axenic culture of Chlorella-bearing *Paramecium bursaria* in the light. *Trans. Am. Microsc. Soc.* 94 109–117. 10.2307/3225536 1114583

[B31] Whitman W. B. Rainey F. Kämpfer P. Trujillo M. Chun J. DeVos P. et al.. (eds). (2015). *Bergey’s Manual of Systematics of Archaea and Bacteria.* Hoboken, NJ: Wiley. 10.1002/9781118960608

[B32] Wichterman R. (1949). The collection, cultivation, and sterilization of *Paramecium*. *Proc. Pa. Acad. Sci.* 23 151–180.

