# Supplementary material for: A defined synthetic algal medium enables lettuce-free culturing of unfed Paramecium bursaria while preserving host-associated microbiome composition
Source: Front Microbiol. 2026 May 1;17:1821058. doi: 10.3389/fmicb.2026.1821058 (PMC13175966; doi:10.3389/fmicb.2026.1821058)
Supplement: Supplementary file 1 [file Data_Sheet_1.pdf]

Supplementary Materials

A defined synthetic algal medium enables lettuce-free culturing of unfed *Paramecium bursaria* while preserving host-associated microbiome composition

Yuri Matsushima, Eiko Himi, Masaharu Kitashima, Kohei Ogura, Susumu Kotani, Akiya Hino, Kazuhito Inoue and Hiroshi Hosoya

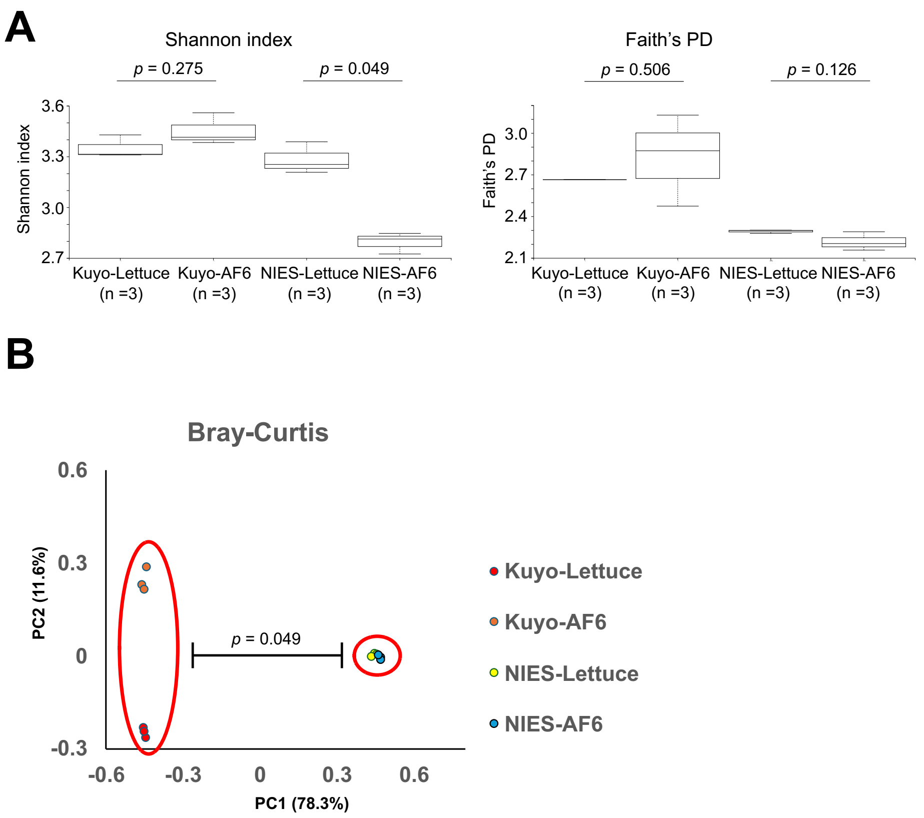

Figure S1. α and β diversity. (A) Shannon index (left) and Faith's Phylogenetic Diversity (Faith's PD) (right). (B) Bray–Curtis distances. Paired-end reads were processed using DADA2 (Callahan et al., 2016) within the QIIME 2 pipeline (Bolyen et al., 2019), with chimeras removed via the "consensus" method. Diversity metrics were calculated at a rarefaction depth of 20,000 reads per sample. Alpha diversity, including the Shannon index and Faith's PD, was compared across groups using the Kruskal–Wallis test. Beta diversity discrepancies were evaluated via Permutational Multivariate Analysis of Variance (PERMANOVA) based on Bray–Curtis distances between the Kuyo (n=6) and NIES (n=6) samples.
